# Editorial: Metabolic Regulation of Stem Cells and Tissue Growth

**DOI:** 10.3389/fnmol.2020.625606

**Published:** 2021-01-28

**Authors:** Christian Lange, Francesco Bifari

**Affiliations:** ^1^Center for Regenerative Therapies Dresden (CRTD), Center for Molecular and Cellular Bioengineering (CMCB), Technische Universität Dresden, Dresden, Germany; ^2^Laboratory of Cell Metabolism and Regenerative Medicine, Department of Medical Biotechnology and Translational Medicine, University of Milan, Milan, Italy

**Keywords:** cell metabolism, regenerative medicine, stem cell differentiation and proliferation, nutrient, oxygen, angiogenesis, metabolic fingerprint, metabolic flux analysis

Metabolism is defined as the sum of all life-maintaining biochemical reactions, which intertwine the extracellular microenvironment (i.e., availability of nutrients, such as glucose, and oxygen) with the functional cellular need (for example, cell growth, migration, or differentiation). Metabolic reactions either convert high-molecular substances to generate energy, molecular building blocks for biosynthesis, and signaling molecules (catabolism), or generate novel high molecular bio-molecules (proteins and proteoglycans, carbohydrates, lipids, and nucleotides) from the available low-molecular building blocks and precursors (anabolism). In metazoans, these processes range from digestion, the distribution of the resulting metabolites toward specific organs and tissues toward compartmentalized metabolism on the cellular and sub-cellular level.

Stem cell division, differentiation, and tissue growth are inherently coupled to anabolic growth and production of biomass. Accordingly, cells in different conditions, such as quiescent stem cells, amplifying precursors and differentiated cells, show dramatic changes in their cellular metabolism. Of note, research during the past two decades has shown that the state of cellular metabolism is a determinant of cell fate, rather than being a mere consequence of it (Missiaen et al., 2017). In this perspective, cell metabolism represents the engine of the cells, onto which all the biological stimuli (including growth, survival, and inflammatory signals) converge. Metabolic reactions regulate major determinants of cell fate such as signaling, epigenetic chromatin modification, and direct control of gene transcription (Knobloch and Jessberger, 2017).

Cellular metabolism undergoes continuous and highly dynamic modifications to provide a way to couple the physiological state of the organism with the stem cell activity in different tissues. Current areas of intense investigation provide new and relevant information on how diseases impairing cellular metabolism directly influence stem cell activity and tissue growth, exploiting the ever-growing disease related information on human genetics.

Importantly, physiological parameters such as tissue oxygenation, temperature (in cold-blooded animals), and nutrient availability are now recognized to influence the fate of stem cells via metabolic reactions (Martano et al.) *In vivo*, supplementation of the stem cell niches with oxygen and nutrients is provided by blood vessels. Thus, stem cell activity and cell maturation are tightly regulated with blood vessel development (Karakatsani et al.), for example in the developing and adult brain. During brain development, the ingrowth of vessels into the neocortex promotes neural stem cell differentiation by triggering a cascade of tissue oxygenation, reduced activity of HIF-1α, and blunted glycolytic metabolism that favors the switch toward neurogenesis (Lange et al., 2016). Robust mitochondrial respiration is a metabolic feature of differentiated neurons (Laughlin et al., 1998; Beckervordersandforth et al., 2017) and enhancing oxidative metabolism can improve stem cell neuronal differentiation (Bifari et al., 2020). In adult brain, astrocytes are thought to be more glycolytic and appear to support the high metabolic need of neuronal cells (Knobloch and Jessberger, 2017). Noteworthily, however, by selectively inhibiting mitochondrial transcription and oxidative phosphorylation in astrocytes, Fiebig et al. showed that, under physiological condition, there is increased reactive gliosis in the cortex. Conversely, following photochemically-induced ischemic stroke they observed in the perilesional area, a significant reduction of astrocytes proliferation and an increase of neuronal cell death.

Karakatsani et al. highlighted the many interactions between blood vessels and stem cells in the brain. Similarly to adult stem cells from different tissues (e.g., pancreas, liver, and adipose tissue), neural stem cells reside in close proximity to specialized blood vessels, which are highly organized with unique architecture characterized by dense network of planar, interconnected, and relatively non-tortuous (straight) vessels (Bifari et al., 2017b; Knobloch and Jessberger, 2017; Decimo et al., 2020). Importantly, specialized blood vessels at the NSC niche have partially permeable blood brain barrier (BBB) allowing the access of signals deriving from the blood. Therefore, in addition to supply oxygen and nutrients, these blood vessels of the stem cell niche provide a variety of blood-born soluble factors, including hormones (i.e., prolactin, erythropoietin, insulin, and cortisol) and cytokines [i.e., CCL11 and the transforming growth factor–β (TGF-β) family member GDF11], as well as endothelial cell secreted and membrane-bound signaling molecules involved in stem cell maintenance and differentiation (Karakatsani et al.). Quiescent adult NSCs appear to be hypometabolic and preferentially utilize glycolysis and fatty acid oxidation (FAO; lipolysis) to support their energy needs (Knobloch and Jessberger, 2017). Blood-born derived insulin/IGF-1 signaling in NSCs triggers the PI3K/Akt pathway and its related signaling, which reduced the FoxO transcription factors and activate mTORc1pathway, resulting in increased differentiation (Karakatsani et al.).

Nutrients and blood-born molecules that influence stem cell self-renewal, proliferation, migration, and differentiation represent potential pharmacological targets for stem cell and tissue growth modulation (Banfi et al., 2018). Schmidt et al. showed that glucocorticoid administration negatively impacts fin regeneration in zebrafish and leads to impaired expression of selected extracellular matrix components, function of ion transporting ATPases and proteins involved in macromolecule and vesicular transport mechanisms. Similarly, Swaminathan et al. provide evidence that the flavonoid derived nutrient luteolin specifically inhibits cell proliferation and decrease neuronal differentiation of embryonic stem cells, whereas it does not inhibit expression of meso- and endodermal markers. The authors showed that lutein directly acts on p300, which mediated specific histone acetylation inhibition, further confirming the potential interaction between cellular metabolites and epigenetic modification (Swaminathan et al.).

Modulation of cell metabolism has been a recognized approach to change cell phenotype and therefore to drive specific cell function. Therefore, basic research data on the metabolic control of stem cells and tissue growth at the cellular, tissue and organismal level during development, disease and regeneration, are providing novel and relevant pharmacological targets, which can be potentially modulated for therapeutic purposes (Bifari et al., 2017a; Missiaen et al., 2017; Martano et al.).

However, the metabolic heterogeneity of cells in a tissue, and the fast and dynamic metabolic modification within the cell, represent major challenges for the exploitation of cell metabolism as a relevant therapeutic target.

Martano et al. summarized the different approaches that can be used for studying stem cell metabolism. The large-scale identification and quantification of the metabolite abundance present at a given time inside the cell (whole metabolome) provide an accurate description of the metabolic state of the sample (metabolic fingerprint). Complementary to the metabolic fingerprint, the investigation of targeted subset of metabolites possibly involved in a specific pathway, and of the metabolic dynamics, i.e., metabolic flux analysis, provides a quantification of the metabolic reactions.

The Heterogeneity of metabolic regulation within a tissue can be addressed by cell type-specific manipulation of metabolic pathways (Lange et al., 2016; Quaegebeur et al., 2016) as well as by the continuously growing repertoire of single-cell analysis (Martano et al.) Moreover, metabolic cell tracking can be performed, based on the characteristics of the probes or vehicles used, applying MRI (magnetic resonance imaging), MRS (magnetic resonance spectroscopy) or PET (positron emission tomography), and SPECT (single photon emission tomography). To study real-time metabolism at a single cell level in living samples, fluorescent metabolic biosensors that are able to bind functional state to a fluorescence protein activity have been also developed (Martano et al.).

Several techniques are now available to study the cell metabolism and investigate how nutrients, drugs, and cell-cell interactions may modulate the metabolic state within the cells. Notably, such metabolic modulation will eventually lead to a modification of the cellular phenotype and potentially a promising way to improve stem cell function and tissue growth in regenerative medicine.

## Author Contributions

CL and FB wrote the manuscript. Both authors contributed to the article and approved the submitted version.

## Conflict of Interest

The authors declare that the research was conducted in the absence of any commercial or financial relationships that could be construed as a potential conflict of interest.
